# Labor and Related Injuries among Schoolchildren in Palestine: Findings from the National Study of Palestinian Schoolchildren (HBSC-WBG2006)

**DOI:** 10.1155/2014/729573

**Published:** 2014-04-03

**Authors:** Christine Jildeh, Ziad Abdeen, Haleama Al Sabbah, Christopher Papandreou, Ibrahim Ghannam, Nancy Weller, Anastas Philalithis

**Affiliations:** ^1^Department of Social Medicine, School of Medicine, University of Crete, P.O. Box 2208, 71003 Iraklion, Crete, Greece; ^2^Faculty of Medicine, Al-Quds University, The Al-Quds Nutrition and Health Research Center, Abu Dies, West Bank, P.O. Box 20760, Jerusalem, Palestine; ^3^Faculty of Medicine, Al-Quds University, P.O. Box 51000, Jerusalem, Palestine; ^4^Faculty of Medicine, An-Najah National University, P.O. Box 7, Nablus, West Bank, Palestine; ^5^Department of Nutrition & Dietetics, Harokopio University of Athens, 70 El. Venizelou Avenue, Kallithea, 17671 Athens, Greece; ^6^Faculty of Nursing, University of Texas, 6901 Bertner Avenue, Houston, TX 77030, USA

## Abstract

*Background*. Labor related injuries among Palestinian schoolchildren are a significant undocumented public health concern. This study aimed at documenting the prevalence and nature of work related injuries among schoolchildren as well as identifying sociodemographic factors that predict these injuries. *Methods*. A cross-sectional survey included 15,963 children of whom 6458 (40.8%) completed an optional package related to labor. Students aged 12–18 years self-completed the international WHO collaborative HBSC valid questionnaires between April and May of 2006. *Results*. Approximately 73.8% of the students who filled the optional package reported working during the last 12 months, of whom 79.1% sustained a work related injury. Work injuries were significantly higher among boys, younger children, and children enrolled in UNRWA schools and living in Gaza Strip (*P* < 0.05). Children working ≥3 hours/day were more likely to experience injuries, 1.73 (95% CI, 1.53–1.95), than those working ≤3/day. About half of the children worked in retail trade (51.5%), agriculture (20.0%), and cleaning (11.4%). Injury type was related to the type of work performed. *Conclusions*. The high prevalence of injuries among working Palestinian schoolchildren confirms its severity as a public health problem. To reduce occupational injuries, policymakers and professionals should develop intervention programs that target the public and health providers.

## 1. Introduction

Child labor is prevalent worldwide [1, 2]. Schoolchildren who work are particularly vulnerable because their roles within various institutional structures are often subject to dramatic developmental shifts and normative crises. Family, school, and work subject adolescents to differing and sometimes contradictory degrees of autonomy, responsibility, purposefulness, and inadequate adult supervision [3].

Young workers run a higher risk of work injuries arising from lack of experience, limited awareness of existing or potential risks, or immaturity [4]. Working methods, tools, and equipment are normally designed for adults and do not take into account the smaller body size of the child worker. Thus, children and young people are at a greater risk of fatigue, injury, and accidents because of ill-fitting tools and safety equipment [5].

Although there is enough research evidencing socioeconomic disparities as a risk factor for unintentional injuries among adolescents (i.e., pedestrian, recreational) [6–11], very few have considered whether such disparities exist in work related injuries [12]. In examining individual level risk factors, studies have found that disparities exist by gender and age [13] as well as ethnicity [14].

A household survey conducted by the Palestinian Center Bureau of Statistics in 2000 estimated the number of children within the 10–17 age group at 605,409. The percentage of documented employed children was 5.5% (*n* = 33,298), of whom 38.4% were not enrolled in schools [15]. The study indicated that 24.0% of these youth worked in the commercial sector including restaurants, 20.0% in mining, quarries, and manufacturing industries, 30.4% in agriculture, and 18.0% in construction. The percentage of children who worked for more than 6 hours a day was 73.2% [15].

Currently, it is estimated that about 40,000 children under 18 are engaged in some form of labor activity in Palestine [16]. The Palestinian Labor Law, effective since mid-2000, sets the age for admission to employment at 15 years and stipulates a number of restrictions on work for children 15–18 years old [17, 18]. However, since the outbreak of the Intifada (uprising) in September 2000, the socioeconomic situation has deteriorated greatly, presumably giving rise to increased child labor. The concept of child labor itself was considered by some of the families and the children as a “positive form of solidarity,” rather than a violation of the rights of the child [19]. Some employers even prefer students as employees because their age/inexperience makes them less paid, more obedient, easier to manage, and less aware of their rights. They also escape from the requirement to pay employment insurance when they employ juveniles.

Little information exists on injury and factors associated with injury among Palestinian working schoolchildren. The lack of national data regarding working schoolchildren aged 12–18 years prompted this study to document the prevalence and nature of work related injuries among children as well as to identify sociodemographic factors that predict these injuries.

## 2. Methods

### 2.1. Participants

The selected sample was stratified according to region (West Bank and Gaza Strip), school types (government, private, and UNRWA), and grades (6th, 8th, 10th, and 12th) and it excluded school-aged children who did not attend school. Assuming an average class size of 35 students, the aim was to recruit 4000 students at each grade level, from both regions, to produce a sample size of around 16000 students. A two-stage cluster sampling technique was used with the school as the primary sampling unit. In the first stage, 405 schools were selected randomly with probability proportional to their size (size defined as number of classes in the school). The list of the schools and number of classes in each school, for the academic year 2005/2006, was obtained from the Ministry of Education. All of the randomly selected schools (*n* = 405) agreed to participate. The response rate was 97.3% among 16400 students who participated on data collection, leaving a sample size of 15963 of whom 6458 (40.8%) completed the optional package related to labor. In the second stage, one class was selected at random from each school. Each of these classes was defined as a cluster, where all the students in the selected class were eligible for inclusion. The aim was to survey one class per school. However, in schools where boys and girls were studying in separate classes, two classes were selected, one for boys and another for girls. This resulted in a total of 481 classes being selected overall.

### 2.2. Instruments

The questionnaire was developed using the WHO international HBSC questionnaire (2001-2002) including all mandatory HBSC questions [20]. The questionnaire was translated into the native language (Arabic) and changes in wording were made to clarify the meaning of certain questions; however, in order to maintain comparability with other countries, the questionnaire changes were kept to a minimum. It was tested in two independent preliminary studies using in-class administration as well as focus-group discussions to demonstrate reliability and validity prior to the administration of the national representative full survey.

In addition to the major variables addressed in the survey (demographics, general health, well-being, family and peer relationships, school environment, lifestyle, exposure to political violence, and mental health) [21–23], the questionnaire contained additional optional packages: half of the optional components of the questionnaire (Form A) contained questions on violence, injuries, and social inequalities and the other half of the questionnaire (Form B) contained optional questions on lifestyle, mental, labor, and physical health. Equal numbers of Form A and Form B were randomly distributed in each school class. However, the injury filter items were asked to all sampled students. This paper presents data from 6458 questionnaires (Form B) which included the mandatory HBSC questions as well as optional questions on lifestyle, mental health, labor, and physical health.

*Sociodemographic Characteristics.* They included individual factors, such as gender, grade (6th, 8th, 10th, or 12th), region (West Bank or Gaza Strip), school ownership (government, private, or UNRWA), family affluence scale (FAS) (low, moderate, and high) [24], and parental education level (low and high).

*Work Intensity*. Work intensity was <3 hours/day and ≥3 hours/day.

*Primary Labor*. Primary labor included (a) agricultural work, (b) retail trade, (c) street traders (selling newspapers, magazines, beverages, and sweets), (d) tool maintenance work (repairing equipment or tools), (e) cleaners, (f) movement of goods, (g) work in building construction and maintenance, (h) workshops like carpentering, mechanics, and aluminum, and (i) house work.

*Work Shift*. Work shift included (a) during school hours, (b) after school hours, (c) on the weekend, and (d) in the semester or annual vacations.

*Types of Injuries*. The occurrence of injury was measured using a question: “did any of the following happen to you in the previous 12 months because of your work?,” and the types of injuries included (a) pain in the back, (b) muscle pain, (c) injuries, deep scratches, or wounds, (d) bone fractures, (e) eye injuries, (f) exposure to poisonous or burn-causing materials, and (g) other. More details regarding the types of injuries were found in a previously published article [25].

### 2.3. Procedure

Selected schools were informed about the survey by sending a letter to the school principle. All school principals agreed to participate. In each school, the student advisor distributed the questionnaires in the classroom and was instructed to respond to the students' enquiries about the procedure. School children completed the questionnaires independently during one school period, lasting 45 minutes. The survey was conducted through self-completed questionnaires and data were collected anonymously in the West Bank and Gaza Strip by Al-Quds Nutrition and Health Research Institute (ANAHRI) in Al-Quds University between April and May of 2006. The 2006 Palestinian HBSC survey was approved by Al Quds University Ethical Committee and the Research Ethics Board of the Palestinian Ministry of Education.

### 2.4. Data Analysis

Data analysis was performed using the SPSS statistical package version 20 (SPSS Inc., Chicago, IL, USA). Chi-square test was used to compare differences in work status, type of work, and related injuries among adolescents by several sociodemographic characteristics. Univariate and multivariate logistic regression models were used to assess the association between work injury and work intensity, work shift, type of work, and sociodemographic characteristics. Crude and adjusted odds ratios (OR) with 95% confidence intervals (CI) were derived. A significance level of *P* < 0.05 was used.

## 3. Results

### 3.1. Sociodemographic Characteristics

The average age of the schoolchildren was 15.1 years; 28.4% of them were in grade 6, 26.6% in grade 8, 24.0% in grade 10, and 20.9% were in grade 12. More than half of the working children resided in the West Bank (55.4%) while the rest resided in Gaza Strip (44.6%). A high percentage of parents whose children were employed had low educational levels (70.9% of the fathers and 64.4% of the mothers). Of all respondents, 73.8% (*n* = 4765) reported working during the last 12 months (private family business 25.2%, in houses 50.6%, in an organization 3.9%, in industrial areas 3.0%, in farms 3.1%, in building workshops 2.8%, in a cafeteria/shop/market 1.4%, in the streets 0.9%, and different varied places 9.1%) and more than two thirds of them were boys (70.9% boys versus 29.1% girls).

Approximately half of the children (52.8%) reported working ≥3 hours/day while 47.2% were employed for <3 hours/day. High work intensity was significantly higher among boys than girls (57.4% versus 41.7% resp.; *P* < 0.05). From all working children, 79.1% reported an occupational injury (boys 81.7% versus girls 73.0%; *P* < 0.05). About one quarter of boys (26.8%) reported current/recent employment after school hours and it was 22.7% for girls. This trend was observed in working during school vacations holidays (21.3% boys versus 14.3% girls, resp.) (*P* < 0.05). The highest prevalence of injuries was noticed among 6th grade students (82.6%) (*P* < 0.05), children from UNRWA schools (82.3%) (*P* < 0.05), and those living in Gaza (81.6%) (*P* < 0.05). More students from 8th grade reported working before school hours and on weekends while more students from 6th grade reported working during school hours and students from 10th grade reported working on the summer holidays (*P* < 0.05). A significantly high proportion of children from government schools (47.7%), West bank (49.1%), and poor families (50.1%) reported working less than 3 hours per day (*P* < 0.05) while children from private schools (41.5%), Gaza Strip (44.8%), and rich families (39.8%) reported employment 3 hours daily or more (Table 1).

Table 2 indicates that most employed students reported working for pay: 51.5% of the students worked in retail trade (boys 46.0% versus girls 61.5%) and 20% worked in agriculture (boys 22.9% versus girls 14.2%); students working as cleaners were 3.3% for boys versus 2.9% for girls (*P* < 0.05). More boys than girls worked in construction/mechanical or maintenance (3.4% boys versus 1.0% girls), followed by street traders (7.3% boys versus 5.7% girls), movement of goods (3.7% boys versus 1.2% girls), and workshops (2.0% boys versus 0.7% girls) (*P* < 0.05), while the contrary happened in concerning house work (6.9% boys versus 10.8% girls; *P* < 0.05). Street traders came mainly from the 8th grade (8.9%; *P* < 0.05), while those working in agriculture production from 6th (21.0%) and 12th grades (21.0%) (*P* < 0.05). More students from governmental schools were employed in the agricultural production (20.5%) while more students from private schools worked in building construction/maintenance (5.1%) and workshops (3.3%) (*P* < 0.05). More children from the West Bank were involved in agricultural production (21.0%), house work (9.3%), building construction/maintenance (3.1%), and workshops (1.9%), while children from Gaza were more involved in retail trade (54.9%), tool maintenance work (3.6%), and movement of goods (3.0%) (*P* < 0.05). In both West Bank and Gaza a significantly (*P* < 0.05) higher proportion of schoolchildren from poorer families worked in retail trade (53.8%) and house work (9.3%), whereas those from richer families worked in agricultural production (23.3%), building construction (3.6%), and workshops (1.7%).

Table 3 presents the type of work injury in relation to type of work. Back pain was reported in higher frequency among working schoolchildren (33.0%) during their participation in agricultural production, muscle pain during construction/mechanical or maintenance (19.8%), and deep scratches or wounds during tool maintenance work (20.7%) (*P* < 0.05). Higher proportions of bone fractures (16.2%) and eye injuries (13.2%) were found among cleaners (*P* < 0.05). Finally, exposure to poisonous or burn-causing materials was found to be more frequently reported among children implicated in workshops (*P* < 0.05).

Table 4 presents the univariate and adjusted odds ratios for work injury by work intensity, work swift, type of work, and sociodemographic characteristics. After controlling for gender, grade, region, parent educational level, and family affluence scale (FAS), schoolchildren working at least 3 hours per day were 1.73 times more likely to get injured than those working less than 3 hours per day. Working during, before, and after school hours as well as during weekend was found to increase the likelihood for injuries compared to working during school vacations (*P* < 0.05). Schoolchildren working as cleaners and in workshops were 0.48 and 0.95 times less likely to get injured than those in agricultural production. Contrarily, children working in retail trade, working as street traders, and involved in house work were 1.61, 2.56, and 1.39 times more likely to get injured, respectively, than those in agricultural production. Boys were 1.56 times more likely to get injured than girls. On the other hand, 8th to 12th grade students had decreased likelihood of injury than their 6th grade counterparts (*P* < 0.05).

## 4. Discussion

### 4.1. Prevalence

According to the authors' knowledge, this is the first national survey to document the labor experiences of Palestinian schoolchildren. More specifically, 73.8% of the students reported being engaged in labor during the past 12 months. Moreover, among Palestinian schoolchildren workers, 47.1% reported working less than three hours daily (less than 15 hours weekly if extrapolated over five work days) and 52.8% more than three hours daily (more than 15 hours weekly extrapolated over a typical work week). Data for 6th, 8th, 10th, and 12th grade Palestinian workers indicate similar patterns. In comparison with data from the National Longitudinal Study of Adolescent Health (Add Health) in 1996 which indicated that less than 8% of 10th graders, 20% of 11th graders, and 46% of 12th graders worked more than 19 hours per week during the school year [13]. Other studies also report similar findings [26–29] including an Indian study reporting that half of working children are employed for less than five hours daily [30]. Discrepancies between these estimates are obvious and may reflect different reference periods and interview protocols, self-versus proxy response, survey design (personal visit versus telephone survey administration, etc.), respondent bias, errors in recall, and other measurement and methodological issues. While estimates of the prevalence and intensity of work vary considerably across samples, this study clearly indicates that substantial numbers of Palestinian youth combine school and part-time employment.

The high prevalence observed among Palestinian working children may be due to the high overall unemployment rate observed in the West Bank (44%) and in the Gaza strip (72%) populations which are below the poverty line [31]. Since the second Intifada in September 2000 and despite child labor law restrictions, increasing numbers of school-aged youth have entered the labor force [16]. Under conditions of economic crisis, youth work may be considered a necessity for families whose income has been severely compromised by geopolitical events. Factors affecting it are reductions in foreign aid, factory closures, and cross-border political factors that are mostly beyond the control of the population [19]. One possible explanation is the high density of the refugee population along the Gaza Strip and an increased fertility rate of 4.5 children per woman in Gaza and the West Bank resulting in a high percentage of children under the age of 15 (42.5%) [32].

### 4.2. Work Intensity and Injury

This study found that out of those who reported working, 79.1% (boys 81.7% versus girls 73.0%) suffered from occupational injuries. This prevalence of injuries was higher when compared with Lithuanian school adolescents (59% among boys and 40% among girls) [33] and Nigerian school children (64.5%) [34]. In the U.S. in 2003, a national telephone survey among teens employed in the retail/service industry estimated that 69% of 16- and 17-year-olds were involved in any kind of work (75% of males, 62% of females) [4, 35].

Our findings from this study indicate that youth employed more than three hours daily (high intensity workers) were approximately 1.3 times more likely to experience an occupational injury than students working fewer than three hours a day. Several studies have revealed that extensive employment during the school year may have deleterious consequences on young workers [36–38]. This may be explained by the fact that children working fewer hours weekly have less exposure to injury risks. A similar dose/response effect was found among South Texas students [39, 40]. In this study, middle and high school students working more than 20 hours weekly were 1.8 and 1.5 times, respectively, more likely to be injured than students working 10 or fewer hours weekly. Methodological issues (daily versus weekly work) prevent more direct comparisons of the two studies [39, 40]; however, working more hours during the school year raises health and safety concerns about working students.

### 4.3. Work Settings and Work Injuries

We found that most of the schoolchildren work was of an informal nature in the retail trades, agriculture, and clerical/cleaning positions. By comparison, in the U.S. slightly more than half of 15- to 17-year-olds are employed in the retail sector (restaurants, grocery stores). Another one-quarter of them work in the service industry while slightly more than one-fifth work in agriculture, manufacturing construction, and other trades [41, 42].

In a study of working children in Nigeria, almost half reported street trading while one-quarter worked in retail and another one-quarter in farming [34]. Most children, however, work in agriculture and industry. In 1992 in Pakistan, more than two-thirds of the estimated 1.5 million employed in the carpet industry were children [43]. Indonesian domestic service employs an estimated 5 million children each year [44]. In India, where most working girls are employed as domestics, children are also employed either in shops, workshops, or companies [30].

The present study indicates that Palestinian schoolchildren are more frequently injured while working in retail trades, such as grocery stores, food establishments, street vending (around one half of all injuries), and agriculture (about one-fifth of the total injuries), and in service positions, such as house cleaning and clerical work (11.4%). In the U.S., also, retail trades produce about half of all injuries to working youth followed by service jobs (one-fifth of the total injuries), agriculture, and manufacturing (11%) [45]. Not surprisingly, most injuries occur in those jobs employing the greatest number of working youth, in this case, the retail sector. However, in Brazil, jobs with the greatest risk for injury include domestic work, waiting tables, and brick making and tile work [46].

### 4.4. Gender, Work Intensity, and Work Injuries

Greater numbers of Palestinian boys reported working compared to girls and with more hours daily. These results are not surprising and are supported by the findings of several nationally representative studies conducted in the U.S.; across the board, these investigations note differences in the prevalence and intensity of work by gender [26, 27, 29]. Only one national study in the U.S. in recent years has determined that more females than males work, overall, and that females work more hours weekly than males [46].

Among Indian child laborers, girls began working one-two years before boys. By the age of 15, however, more boys than girls were working and about one-quarter of the boys in this Indian study worked for more than 10 hours daily [30].

Boys appear to be more likely to sustain an injury at work than girls. This study found that Palestinian boys were 1.6 times more likely to be injured than girls. Numerous other investigations of youth work also find an injury rate for boys that is about twice that of girls [35, 37, 47, 48]. In the south Texas study, middle and high school boys were 50 percent more likely to be injured than girls [39, 40]. These findings could partly be explained by the disparity in weekly work hours between the genders and the greater proportion of working males. Different types of jobs worked by Palestinian boys and girls could also be responsible for this difference. For example, Palestinian boys were working in occupations that are known to be hazardous for workers of any age like manual labor, such as agriculture, construction, movement of goods, and street trade, while girls worked in retail stores, service positions, such as cleaning, and clerical jobs [35]. The Institute of Medicine report of 1998 indicates that U.S. boys are more likely to work in manual labor than girls who are more likely to work in service positions [13, 41, 42]. These investigations note that adolescent males frequently work in riskier work situations with greater exposure to work related hazards than females.

### 4.5. Grade and Work Injury

The present study indicated that younger children (6th graders) were more likely to experience a work injury than older ones (8th, 10th, and 12th graders). Although the U.S. national studies and those at the state level showed similar patterns of injury by age, older US children sustained more injuries (85% of all nonfatal work related injuries occur in 16- and 17-year-olds) than younger children[45, 47, 49]. Differences in child labor laws, exposure to hazardous situations, levels of responsibility, and work intensity levels in the two areas may be partially responsible for these variations.

Type of work, however, does not appear to explain this discrepancy as about half of all workers in this study, regardless of grade or age, worked in retail, the category of work least likely to result in a work injury. Another fifth of all workers, again regardless of grade/age, were employed in agriculture, a type of work that frequently produces injuries in workers of any age. We may only theorize that their lack of experience, premature cognitive and developmental systems, undeveloped judgment, more immature psychosocial and emotional development, and the pressure of balancing the demands of school and work could play a role in the incidence of work related injury in these younger youth [50, 51]. Studies of adult workers find that inexperience on the job contributes to occupational injuries [52]. It is plausible, then, that the inexperience of preadolescents and early adolescents turns out to be an important factor contributing to increased injury in these youth. These characteristics of preteens, not coincidentally, constitute the rationale behind child labor laws in many nations that prohibit formal employment in preadolescents until an enhanced age and level of maturity has been attained [13].

### 4.6. Types of Injuries

Among Palestinian adolescents, common reported injuries were strains and sprains (about one-half of all injuries), cuts/bruises/lacerations and fractures, which make up about one-fifth of all injuries, and eye injuries and poisoning, which comprise small percentages of the total injury amount. Similar patterns are seen among U.S. youth where the most common nonfatal injuries in order of decreasing prevalence are lacerations (about one-third of all injuries), contusions or abrasions (almost one-fifth of the total injuries), sprains and strains (slightly less than one-fifth), and burns and fractures (slightly less than one-fifth) [45]. These variations may be accounted for by differences in work characteristics, the work environment, the interaction between work and worker, various types of work, age of working youth, differences in equipment, inadequate job training, health and safety training, supervision patterns, and inappropriate or illegal job assignments [53, 54].

### 4.7. Limitations

Several cautions should be noted in interpreting study results: (1) data represent workers attending school during April-May, 2006, and do not include the large numbers of Palestinian children who are not enrolled in school; (2) results from this study are based on self-reported data and thus are subjected to accuracy of recall; (3) cutoff points for daily work hours were arbitrarily set and limited to the categories used in this analysis (<3 hours/day; >3 hours/day).

## 5. Conclusions

This is the first national study in Palestine to examine the prevalence and characteristics of work related injuries among schoolchildren. Our results both replicate and contradict the findings of other studies on the same topic among very different young populations. In view of our findings and those of other studies, we believe that parents, policymakers, and professionals involved with schoolchildren should carefully monitor the work circumstances of this exceedingly vulnerable population. Increased efforts are needed to reduce labor injuries. Since children need to work to supplement their family income, they must be trained and monitored with special emphasis on the youngest as they are the most vulnerable. Working schoolchildren, parents, employers, medical providers, and school personnel should be targeted for education, training, and counseling about health and safety hazards and safeguards as well as workplace rights and responsibilities for working schoolchildren. More qualitative research is needed for in-depth understanding of the main reasons behind the high percentage of adolescents' labor in order to find out the appropriate interventions to reduce work related injuries.

## Figures and Tables

**Table 1:** Sociodemographic characteristics of adolescents according to work status and related injuries in the Palestinian HBSC-2006 study.

Sociodemographic characteristics					Work shift											
	Low intensity (<3 h/d)		High intensity (≥3 h/d)		During school hours		Before school hours		After school hours		On the weekend		End of school semester/academic year		Work related injuries	
	%	*n*	%	n	%	n	%	n	%	n	%	n	%	n	%	n
Gender																
Boys	**42.6**	1439	57.4*	1941	**16.7**	514	**13.7**	420	26.8*	823	**21.4**	658	21.3*	654	81.7*	2760
Girls	58.3*	807	**41.7**	578	20.8*	240	16.0*	185	**22.7**	262	26.2*	303	**14.3**	165	**73.0**	1011
Grade																
6th grade	**48.1**	650	**51.9**	702	23.1*	274	**15.7**	186	**27.8**	330	**20.3**	241	**13.1**	155	82.6*	1117
8th grade	**48.0**	609	**52.0**	661	**18.5**	207	16.6*	185	**22.1**	247	24.5*	274	**18.3**	204	**76.9**	976
10th grade	**44.5**	510	**55.5**	635	**11.9**	121	**11.7**	119	28.8*	294	**23.5**	240	24.2*	247	**76.3**	874
12th grade	**47.8**	477	**52.2**	521	**16.9**	152	**12.8**	115	**23.8**	214	**22.9**	206	**23.7**	213	**80.6**	804
School ownership																
Gov.	47.7*	1843	**52.3**	2019	**17.7**	607	14.8*	507	**26.0**	890	**22.1**	756	**19.3**	661	**79.5**	3071
UNRWA	**46.2**	279	**53.8**	325	20.0*	108	**14.2**	77	28.3*	153	**22.7**	123	**14.8**	80	82.3*	497
Private	**41.5**	124	58.5*	175	**14.9**	39	**8.0**	21	**16.0**	42	31.3*	82	29.8*	78	**67.9**	203
Region																
West Bank	49.1*	1295	**50.9**	1345	**17.2**	399	**12.4**	289	**25.4**	590	23.1*	536	21.9*	509	**77.1**	2036
Gaza Strip	**44.8**	951	55.2*	1174	18.7*	355	16.6*	316	26.0*	495	**22.4**	425	**16.3**	310	81.6*	1735
FAS																
FAS 1 (low)	50.1*	1421	**49.9**	1417	18.4*	465	**14.6**	369	**25.8**	653	**22.2**	562	**18.9**	478	**79.0**	2243
FAS 2 (moderate)	**43.0**	482	**57.0**	639	**16.2**	163	14.9*	150	26.4*	265	**22.6**	227	**19.8**	199	**80.6**	904
FAS 3 (high)	**39.8**	208	60.2*	315	**14.7**	68	**9.7**	45	**24.6**	114	26.7*	124	24.4*	113	**78.6**	411
Father's education																
Low education	**47.2**	1594	**52.8**	1784	**18.3**	553	**13.6**	410	**26.0**	785	**22.9**	691	**19.2**	581	**79.5**	2686
High education	**45.5**	522	**54.5**	626	**16.3**	165	**15.1**	153	**25.8**	261	**23.3**	236	**19.4**	196	**80.0**	918
Mother's education																
Low education	**47.4**	1457	**52.6**	1614	19.1*	522	15.0*	410	**25.3**	690	**21.9**	597	**18.7**	509	**79.1**	2429
High education	**45.7**	689	**54.3**	819	**14.9**	201	**12.5**	169	27.2*	367	24.4*	329	20.9*	282	**80.5**	1214

*n*: sample size; *P* value of chi-square test.

*Significant at *P* < 0.05.

**Table 2:** Sociodemographic characteristics of adolescents according to type of work in the Palestinian HBSC-2006 study.

Type of work	Gender		Grade				School ownership			Region		FAS		
	Boys	Girls	6 G	8 G	10 G	12 G	Gov.	UNRWA	Private	West Bank	Gaza Strip	FAS 1 (low)	FAS 2 (moderate)	FAS 3 (high)
Agricultural production														
%	22.9*	14.2	21.0*	17.8	19.9	21.0*	20.5*	15.7	19.8	21.0*	18.3	18.1	21.2	23.3*
*n*	966	317	365	307	321	290	1076	129	78	781	502	714	317	163
Retail trade														
%	46.0	61.5*	49.3	52.7	53.7*	50.1	51.3	54.4*	47.3	48.9	54.9*	53.8*	49.1	47.9
*n*	1948	1377	857	908	868	69.2	2691	448	186	1818	1507	2120	732	336
Street traders														
%	7.3*	5.7	7.1	8.9*	5.0	5.7	6.8	7.2*	5.6	6.7	6.9	6.4	6.8*	6.4
*n*	307	128	123	153	81	78	354	59	22	247	188	254	102	45
Tool maintenance work														
%	4.3*	2.0	4.1*	3.3	2.9	3.8	3.5	3.8*	3.8*	3.4	3.6*	3.0	4.6*	3.3
*n*	182	45	72	56	47	52	181	31	15	127	100	119	69	23
Cleaners														
%	3.3*	2.9	4.8*	3.2	2.2	2.2	2.9	5.0*	3.3	2.9	3.6*	2.7	4.0*	2.7
*n*	138	66	84	55	35	30	150	41	13	106	98	107	59	19
Movement of goods														
%	3.7*	1.2	2.9	2.4	3.7*	2.4	2.8	2.7	4.3*	2.7	3.0*	2.8	3.4*	2.7
*n*	158	27	51	41	60	33	146	22	17	102	83	111	50	19
Building construction/maintenance														
%	3.4*	1.0	2.1	2.0	3.3*	3.0	2.4	2.3	5.1*	3.1*	1.9	2.3	2.7	3.6*
*n*	145	22	36	35	54	42	128	19	20	116	51	91	41	25
Workshops like carpenting, mechanics, aluminum														
%	2.0*	0.7	1.0	1.9*	1.5	1.9*	1.5	1.2	3.3*	1.9*	1.1	1.4	1.7*	1.7*
*n*	84	16	17	32	25	26	77	10	13	70	30	55	25	12
House work														
%	6.9	10.8*	7.8	7.8	7.7	9.9*	8.4*	7.8	7.4	9.3*	6.7	9.3*	6.5	7.7
*n*	290	242	135	135	125	137	439	64	29	347	185	367	97	54

*n*: sample size; *P* value of chi-square test.

*Significant at *P* < 0.05.

**Table 3:** Type of work according to type of injury in the Palestinian HBSC-2006 study.

Type of work	Type of injury													
	Back pain		Muscle pain		Injuries, deep scratches, or wounds		Bone fractures		Eye injuries		Exposure to poisonous or burn-causing materials		Other, specify	
	*n*	%	n	%	n	%	n	%	n	%	n	%	n	%
Agricultural production	423	33.0*	178	13.9	137	10.7	92	7.2	66	5.1	30	2.3	117	9.1
Retail trade	814	24.5	398	12.0	317	9.5	213	6.4	141	4.2	69	2.1	472	14.2
Street traders	72	17.7	49	11.3	35	8.0	21	4.8	22	5.1	5	1.1	66	15.2
Tool maintenance work	45	19.8	30	13.2	47	20.7*	29	12.8	21	9.3	6	2.6	13	5.7
Cleaners	43	21.1	28	13.7	27	13.2	33	16.2*	27	13.2*	8	3.9	19	9.3
Movement of goods	47	25.4	33	17.8	25	13.5	18	9.7	14	7.6	7	3.8	20	10.8
Building construction/maintenance	42	25.1	33	19.8*	14	8.4	15	9.0	10	6.0	4	2.4	20	12.0
Workshops like carpenting, mechanics, aluminum	19	19.0	15	15.0	20	20.0	10	10.0	8	8.0	5	5.0*	7	7.0
House work	158	29.7	59	11.1	34	6.4	29	5.5	11	2.1	10	1.9	97	18.2*

*n*: Sample size; *P* value of chi-square test.

*Significant at *P* < 0.05.

**Table 4:** Crude and adjusted^‡^ odds ratios (95% confidence intervals) for the association of work related injuries with work intensity, work swift, type of work, and sociodemographic characteristics in the Palestinian HBSC-2006 study.

	Crude OR	95% CI	Adjusted OR	95% CI
Work intensity				
h/d	1		1	
≥3 h/d	1.73*	1.53–1.95	1.30*	1.11–1.54
Work shift				
End of school semester/academic year	1		1	
During school hours	2.48*	1.93–3.18	2.38*	1.80–3.12
Before school hours	2.34*	1.77–3.09	2.20*	1.63–3.01
After school hours	1.76*	1.43–2.16	1.55*	1.30–1.94
On the weekend	1.52*	1.25–1.85	1.55*	1.31–1.90
Type of work				
Agricultural production	1		1	
Retail trade	1.61*	1.37–1.89	1.61*	1.35–1.92
Street traders	2.53*	1.98–3.21	2.56*	1.96–3.34
Tool maintenance work	0.82	0.56–1.20	0.77	0.49–1.19
Cleaners	0.44*	0.27–0.73	0.48*	0.28–0.83
Movement of goods	0.55*	0.34–0.89	0.69	0.42–1.12
Building construction/maintenance	0.91	0.59–1.39	1.01	0.64–1.59
Workshops like carpenting, mechanics, aluminum	0.83	0.47–1.44	0.95*	0.53–1.71
House work	1.46*	1.15–1.86	1.39*	1.07–1.80
Gender				
Girls	1		1^1^	
Boys	1.87*	1.66–2.10	1.56*	1.32–1.84
Grade				
6th grade	1		1^2^	
8th grade	0.65*	0.55–0.76	0.52*	0.41–0.65
10th grade	0.62*	0.53–0.73	0.48*	0.38–0.60
12th grade	0.79*	0.66–0.94	0.58*	0.46–0.74
FAS				
FAS 1 (low)	1		1^3^	
FAS 2 (moderate)	0.86*	0.79–0.93	0.93	0.85–1.02
FAS 3 (high)	0.85*	0.75–0.95	0.94	0.82–1.06
Region				
West Bank	1		1^4^	
Gaza Strip	1.22*	1.08–1.37	1.15	0.99–1.35
Father's education				
Low education	1		1^5^	
High Education	0.97	0.89–1.06	1.02	0.92–1.12
Mother's education				
Low education	1		1^5^	
High education	0.98	0.82–1.18	0.95	0.80–1.10

CI: confidence intervals; OR: odds ratio; FAS: family affluent scale.

*P < 0.05.

^‡^Adjusted for gender, grade, region, parent education, and FAS.

^1^Adjusted for grade, region, and parent education.

^2^Adjusted for gender, region, parent education, and FAS.

^3^Adjusted for gender, grade, region, and parent education.

^4^Adjusted for gender, grade, parent education, and FAS.

^5^Adjusted for gender, grade, region, and FAS.
